# Simulated microgravity-mediated reversion of murine lymphoma immune evasion

**DOI:** 10.1038/s41598-019-51106-y

**Published:** 2019-10-10

**Authors:** Jillian H. Bradley, Shannon Barwick, Gillian Q. Horn, Elizabeth Ullrich, Brianna Best, Jennifer P. Arnold, Randal K. Gregg

**Affiliations:** 1 Magnolia Research Center, Division of Biomedical Sciences, Department of Microbiology and Immunology, Edward Via College of Osteopathic Medicine Carolinas Campus, Spartanburg, SC 29303 USA; 2 Department of Biology, Chemistry and Physics, Converse College, Spartanburg, SC 29302 USA; 3 Department of Cellular Biology and Anatomy, Augusta University, Augusta, GA 30912 USA; 4 Department of Basic Medical Sciences, DeBusk College of Osteopathic Medicine at Lincoln Memorial University – Knoxville, Knoxville, TN 37932 USA

**Keywords:** T-cell lymphoma, Immune evasion, T-helper 1 cells, Cytotoxic T cells, Immunoediting

## Abstract

No human has returned to the moon since the end of the Apollo program 47 years ago, however, new missions are planned for an orbital outpost. Space radiation and the potential for cancer remain as important issues to the future of human space exploration. While improved shield technologies and protective biologicals are under development, little is known concerning the interaction between cancer cells and host immunity in microgravity. As a hallmark of cancer, tumor cells employ mechanisms of immune evasion to avoid elimination by protective CD4^+^ and CD8^+^ T cells. We showed that a murine lymphoma was able to produce a soluble factor that inhibited the function of dendritic cells in activating the CD4^+^ T cells. Culture of the lymphoma cells in simulated microgravity (SMG), and not Static conditions, restored the CD4^+^ T cell response and augmented CD8^+^ T cell-mediated destruction of the cancer cells *in vitro* and *in vivo*. Thus, SMG impaired the mechanism of tumor escape and rendered the cancer cells more susceptible to T cell-mediated elimination. The stress of microgravity may expose the most critical components of a tumor’s escape mechanism for astronaut protection and the generation of new cancer therapeutics for patients on Earth.

## Introduction

The Artemis program of the National Aeronautics and Space Administration (NASA) is the agency’s first step to extend human missions from low Earth orbit aboard the International Space Station to the Moon by 2024. The establishment of the Gateway orbital outpost around the Moon will allow astronauts to live and work upon arrival via the Orion spacecraft. Given the ultimate goal of human exploration of Mars, the Artemis program will help astronauts learn about living and operating on another world and will lead to the development of technologies needed for long-term spaceflight to and formation of residence on the red planet. However, one of the biggest challenges facing human space exploration is cosmic radiation and solar particles^1^. While there are shielding technologies in use and more in development, the lack of a protective magnetic shield, such as that provided to astronauts aboard the ISS by the Earth, could put humans of long-duration spaceflight at risk for cellular damage and transforming events leading to cancer.

Immune dysfunction is a clear consequence of spaceflight microgravity, however, some type of reduced host response remains intact as a number of astronauts remain relatively healthy during and following spaceflight^2^. Indeed, our lab showed that protective immune T cells declined in response to peptide as a consequence of increasing time cultured in simulated microgravity (SMG) conditions generated by a rotary cell culture system (RCCS)^3^. While work continues to illuminate immune deficiencies in microgravity and SMG, little is known concerning how tumors behave in these environments. Cancer remains one of the leading causes of death worldwide with the number of cases expected to increase to 20 million in the next 10 years^4^. The cancer cells interact with cells of the immune system throughout the process of tumor development and metastasis with the balance of such interfaces representing the major determinant of clinical outcome. A hallmark of cancer is the capacity of the transformed cells to evade the mechanisms of immune control and eradication^5^.

Due to the genetic instability of cancer cells, a tumor is a collection of heterogeneous cells in which some are more recognizable by immune cells than others. Cancer cells that are recognized by the CD4^+^ and CD8^+^ T cells can be eliminated. Other cancer cells express mechanisms to evade immunity, including T cell killing, resulting in tumor escape and clinical disease. The evading cancer cells have been shown to utilize two modes of escape involving loss of recognition molecules or the formation of an immune-suppressive microenvironment^6^. First, cancer cells produce proteins for growth and maintenance as do non-transformed cells of the body. Peptides generated from cellular proteins are loaded onto major histocompatibility complex (MHC) class I molecules and the MHC I-peptide displayed on the cell surface. CD8^+^ T cells possess a T cell receptor (TCR), which upon ligation with the displayed peptide, release compounds such as interferon-gamma (IFN-γ) and cytotoxins that induce apoptosis in the target cells. Peptides that promote such a response are often mutant versions or overexpressed wild-type peptides that are normally restricted to the intracellular space. Thus, the expression of immunogenic peptides by a cancer cell triggers elimination by CD8^+^ T cells. Alternatively, specialized cells called dendritic cells (DC) survey the tissues for proteins. Upon uptake, the proteins are enzymatically digested into peptides and loaded onto MHC class II molecules that can be recognized by CD4^+^ T cells. Upon TCR engagement, the CD4^+^ T cells expand and produce growth factors such as interleukin-2 (IL-2) and immune regulating proteins (or cytokines) that promote a vast array of cellular activities to destroy the source of the peptide. IL-2 is also a critical factor for CD8^+^ T cell expansion and activation^7^. Peptides and peptide-loaded DC travel from the tissues through extracellular fluids (lymph) to lymph nodes where T cells are activated by DC presentation of their respective peptides. Activated T cells then leave the lymph nodes and migrate to the tissue where the cancerous or infected cells reside. Upon arrival, CD8^+^ T cells locate and destroy the diseased cells, as discussed above, while CD4^+^ T cells promote inflammation and activate additional cells against the targets. Tumor evasion in this regard involves loss of MHC class I molecule expression on the cell surface or reduced production of tumor cell-derived peptides^8^. Additionally, tumors can recruit immune cells (i.e. regulatory T cells) that usually operate in preventing autoimmune disease or dampening inflammation^9^.

Given the impairment documented in immunity when exposed to SMG^2^, we hypothesized that SMG would diminish the evasiveness of cancer cells. E.G7-OVA (E.G7), a chicken ovalbumin protein (OVA)-expressing lymphoma cell line, was used to test this proposal. The E.G7 cancer cells were able to suppress dendritic cell activation of OVA-specific CD4^+^ T cell IL-2 production relative to T cell activation in the absence of E.G7. Inhibition of dendritic cell function was mediated by a soluble factor released by the E.G7 cells. This is the first study to identify a tumor evasion mechanism in the lymphoma line which did not employ commonly defined escape mechanisms. Furthermore, while E.G7 cancer cells promoted the activation of peptide-specific CD8^+^ T cells, SMG exposure of the E.G7 targets significantly increased both T cell-mediated IFN-γ production and tumor cell lysis. Similarly, CD4^+^ T cell IL-2 production was increased when DC were co-cultured with SMG exposed E.G7 cells. Finally, these findings translated into robust tumor control *in vivo* as mice challenged with SMG cultured E.G7 cells were more protected from tumor formation than animals given Static E.G7 lymphoma cells. Overall, SMG exposure diminished the specific tumor escape mechanism of E.G7 lymphoma cells and led to augmented T cell responsiveness *in vitro* and tumor elimination *in vivo*. The use of SMG further resulted in the detection of the critical mechanism for the aggressiveness of this lymphoma albeit it was not specifically identified. Therefore, this outcome supported the notion that the stress of SMG or microgravity upon cancer cells may reveal relevant pathways critical for tumor resistance to the host response and provide targets for cancer therapeutics for patients on Earth.

## Results

### E.G7 cancer cells suppress DC-mediated activation of CD4^+^ T cell cytokine production

**Figure 1:**
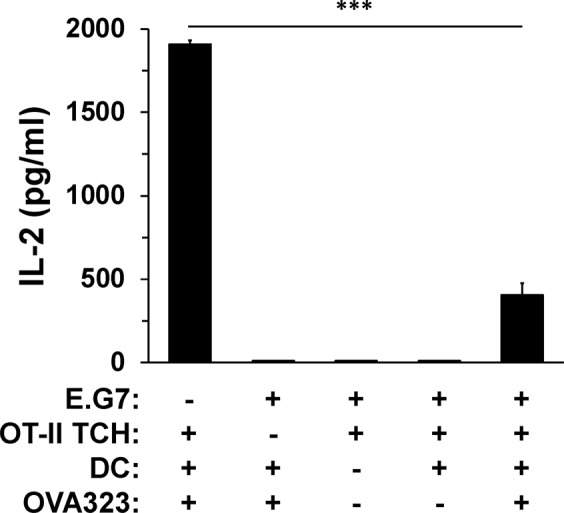
E.G7 lymphoma cells suppress the activation of CD4^+^ T cells. CD4^+^ OT-II TCH (1.25 × 10^5^) were stimulated with JAWS II DC (5 × 10^4^) and OVA323 (0.1 mg/ml) in the presence or absence of E.G7 cancer cells (2.5 × 10^4^) for 24 h (1 ml total volume/well in duplicate). Wells including: E.G7 + DC + OVA323; E.G7 + OT-II TCH; and E.G7 + DC + OT-II TCH served as controls. At the completion of incubation, culture supernatants were collected and assessed for IL-2 production by ELISA. For each bar, the data represent the mean + SD of n = 6 of three independent experiments (n = 18/condition). ***p-value ≤ 0.0001 comparing OT-II TCH IL-2 production in the presence and absence of E.G7 cancer cells.

E.G7 was generated from the parent lymphoma line, EL-4, in order to study OVA peptide-specific CD8^+^ T cell responses to the tumor^10^. Curiously, despite the strong cancer-specific CD8^+^ T cell response, *in vivo* tumor growth was similar to EL-4^11^. To this end, we established an *in vitro* culture system to better understand the interplay between the E.G7 lymphoma cells and activation of tumor-specific T cells by peptide-loaded DC. CD4^+^ T cells were selected for evaluation given their important role in producing IL-2 to support the optimization of tumor lysing CD8^+^ T cells^7^. The murine DC line, JAWS II, was used to activate the CD4^+^ T cells given their expression of MHC class II molecules that bind and display OVA peptide made up of amino acids 323–339 (OVA323). OT-II TCH, a CD4^+^ T cell hybridoma line expressing a TCR that binds OVA323, served as the responding T cells and IL-2 was measured as a readout for cellular activation. Neither E.G7 lymphoma nor OT-II TCH cells constitutively produced IL-2 in the cultures, therefore, any detected cytokine production would represent the outcome of DC presentation of peptide and T cell recognition (Fig. 1). In addition, JAWS II DC, with or without loaded peptide, did not produce IL-2. JAWS II DC were able to capture exogenously added OVA323 for the activation of CD4^+^ OT-II TCH leading to significant production of IL-2. However, a four-fold decrease in IL-2 was observed when E.G7 cancer cells were added to the OVA323-presenting DC and OT-II TCH co-culture (Fig. 1).

**Figure 2:**
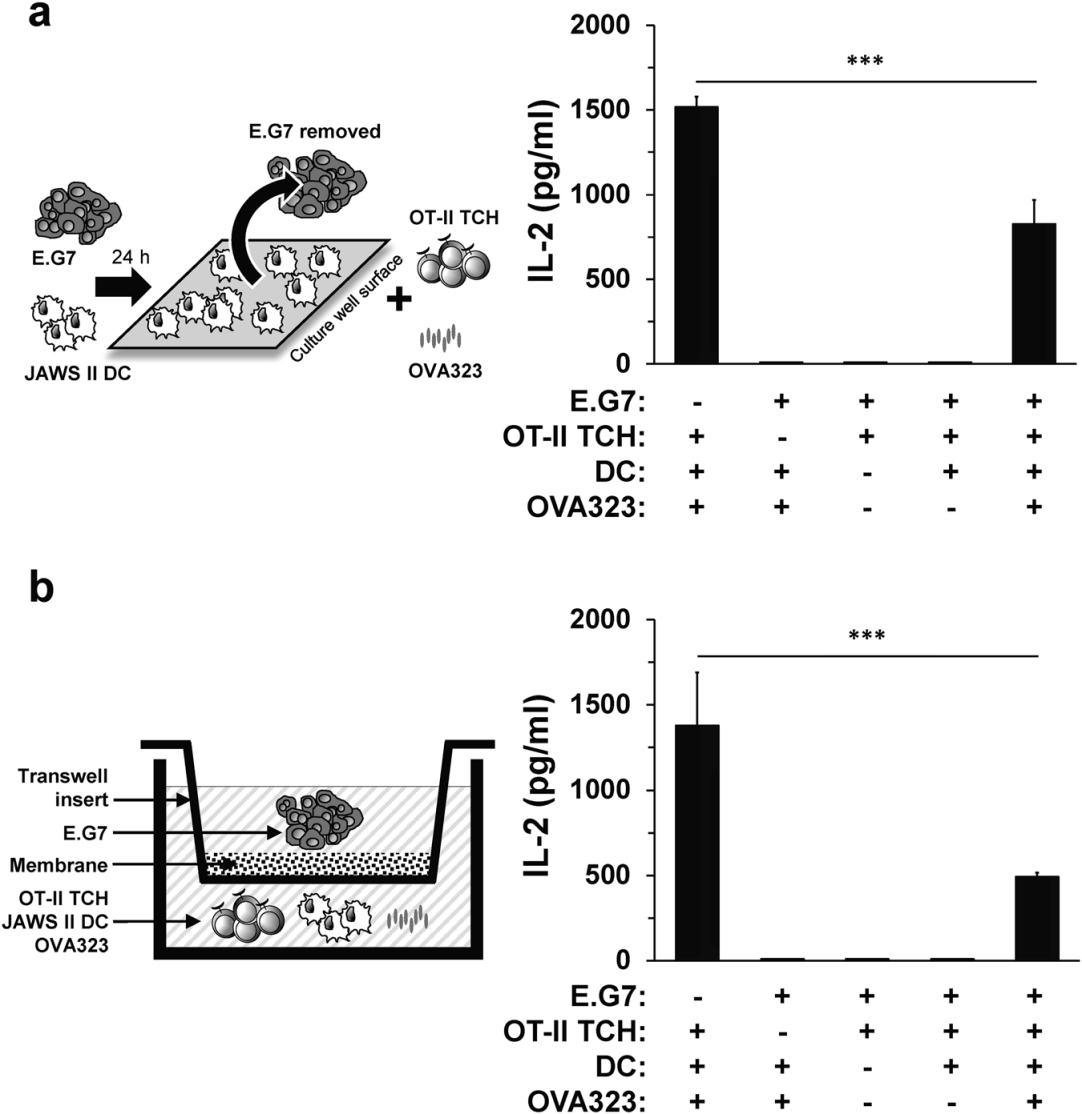
E.G7 cancer cells impair dendritic cell capacity to activate CD4^+^ T cells. (**a**) As shown in the left panel, JAWS II DC (5 × 10^4^) were co-cultured with E.G7 cancer cells (2.5 × 10^4^) for 24 h. 80% of the JAWS II DC added to the culture were adherent to the plate while E.G7 cancer cells remain suspended in the media. Subsequently, the E.G7 cells were removed from the co-culture and replaced with fresh media, CD4^+^ OT-II TCH (1.25 × 10^5^) and OVA323 (0.1 mg/ml) and incubation continued 24 h (1 ml total volume/well in duplicate). In the control wells: E.G7 + DC + OVA323; E.G7 + OT-II TCH; and E.G7 + DC + OT-II TCH, the E.G7 tumor cells were removed and replaced with fresh media prior to the addition of DC, OT-II TCH and/or OVA323. (**b**) As indicated in the left panel, the cells were cultured as in (**a**) with the modification that the JAWS II DC and E.G7 cancer cells were separated by a Transwell insert for the initial 24 h incubation. Afterward, the insert with the E.G7 cancer cells was removed prior to the addition of the OT-II TCH and/or OVA323 antigen. Right panels of both (**a**,**b**) indicate the result of T cell IL-2 production by ELISA. For each bar, the data represent the mean + SD of n = 6 of two independent experiments (n = 12/condition). ***p-value ≤ 0.0001 comparing OT-II TCH IL-2 production when JAWS II DC were cultured in the presence and absence of E.G7 cancer cells.

Given that the peptide was provided exogenously and the T cells required DC for peptide presentation in order to undergo activation, the mechanism employed by the E.G7 cells most likely targeted the DC directly. To address this issue, E.G7 cancer cells were incubated with JAWS II DC prior to the addition of the T cells. After 24 h, E.G7 lymphoma cells were removed and fresh media was added to eliminate any tumor-associated inhibitory ligand or soluble factors from the activation assay (Fig. 2a, left panel). Of note, 80% of the JAWS II DC were adherent to the culture wells while E.G7 cells remained in suspension. Thus, the separation of E.G7 cells from JAWS II DC was straightforward especially upon subsequent washes with fresh media. Despite the removal of the E.G7 cells, T cell production of IL-2 was nearly the same as when E.G7 lymphoma remained in the culture during T cell activation (Figs 1 and 2a, right panel). Therefore, E.G7 cancer cells negatively impacted OT-II TCH activation by suppressing the functions of DC.

Tumors often employ cell-contact and/or secreted factor-dependent inhibitory mechanisms of tumor evasion^6^. To determine whether E.G7 lymphoma cells were using a cell-contact or soluble factor to diminish dendritic cell function, a Transwell insert was included during the pre-incubation of E.G7 cancer cells with JAWS II DC to prevent direct cell-to-cell interaction (Fig. 2b, left panel). Removal of the E.G7 cancer cells did not impact the reduced IL-2 production by the T cells when compared to levels secreted from T cells activated by DC in the absence of the tumor cells (Fig. 2b, right panel). This suggested that E.G7 cancer cells secreted a soluble factor to reduce the activation of the T cells by JAWS II DC.

### SMG exposure diminishes E.G7-mediated evasion of CD4^+^ T cell activation and exacerbates CD8^+^ T cell responses directed against the tumor cells

**Figure 3:**
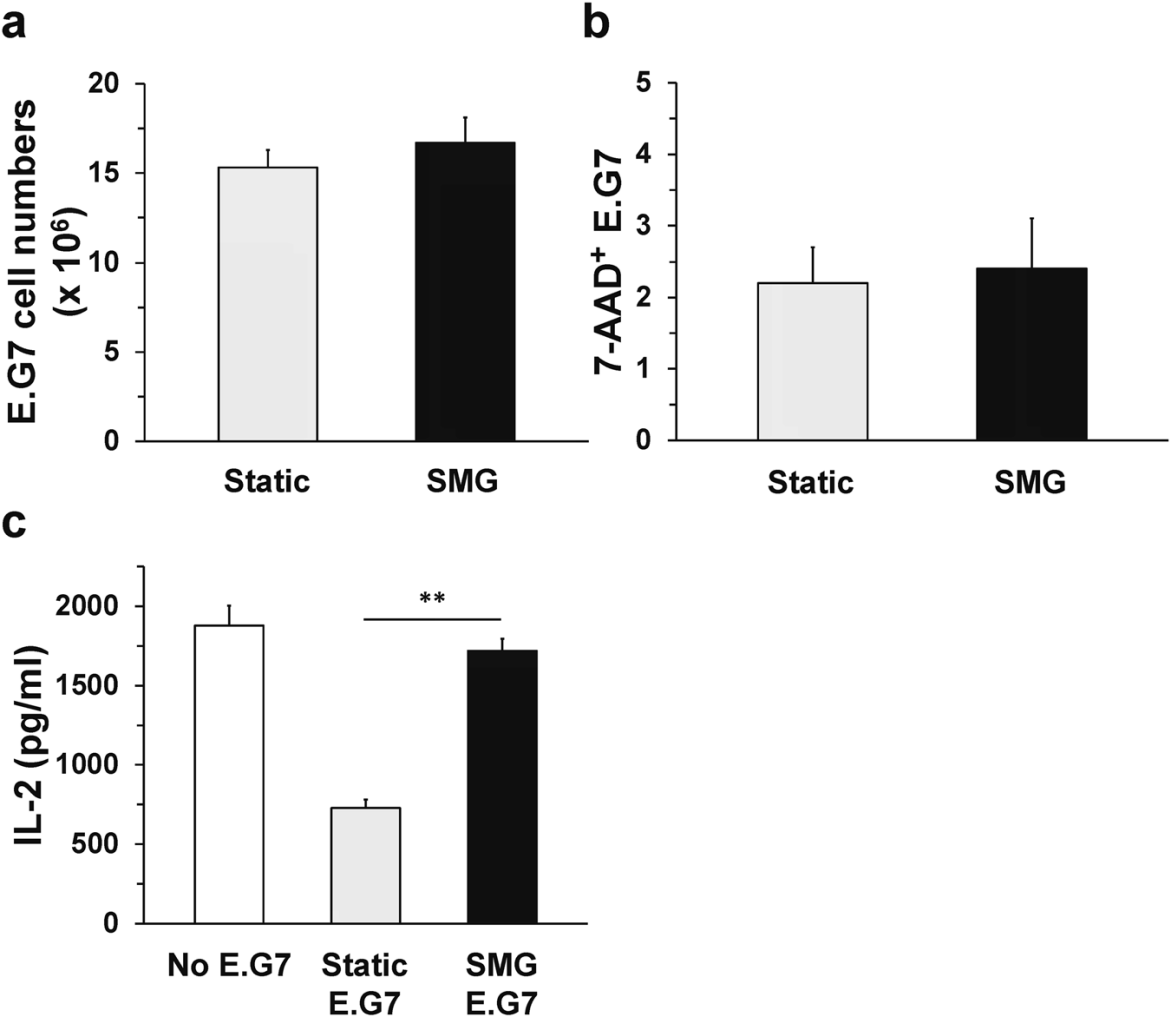
SMG exposure reverses E.G7 tumor-mediated suppression of CD4^+^ T cell activation. E.G7 tumor cells (2 × 10^5^/ml) were cultured in Static (gray bar) or SMG (black bar) conditions for 72 h. (**a**) Following culture, Static and SMG E.G7 were collected and enumerated by Trypan exclusion for the total number of cells in the 10 ml cultures. (**b**) Additionally, the Static and SMG E.G7 cancer cells were stained with 7-AAD and analyzed by flow cytometry for the numbers of 7-AAD^+^ (apoptotic) cells within the Static and SMG E.G7 population (×100%). For each bar, the data represent the mean + SD of three independent, paired cultures. (**c**) Following culture, Static and SMG E.G7 cells were harvested, washed and incubated (2.5 × 10^4^) separately with JAWS II DC (5 × 10^4^) for 8 h. To each co-culture, CD4^+^ OT-II TCH (1.25 × 10^5^) and OVA323 (0.1 mg/ml) were added and incubation continued for 24 h. Control wells were included that contained DC, OT-II TCH and OVA323 antigen (white bar). IL-2 was assessed by ELISA from culture supernatants. For each bar, the data represent the mean + SD of n = 6 of two independent experiments (n = 12/condition). **p-value ≤ 0.001 comparing OT-II TCH IL-2 production when DC exposed to Static and SMG cultured E.G7 tumor cells.

E.G7 cancer cells were not previously shown to secrete a soluble factor to reduce T cell activation. Recently, it was shown that SMG inhibited the growth of Hodgkin’s lymphoma cells due to altered signaling and subsequent mitochondrial dysfunction^12^. Therefore, we hypothesized that SMG would disrupt the mechanism of immune evasion in E.G7 cancer cells and lead to increased responses of CD4^+^ T cells. Before testing this hypothesis, we first evaluated the effect of SMG upon the growth and viability of the E.G7 cancer cells. If the growth of the E.G7 cells is reduced or the cells have lower viability in SMG, compared to Static culture, then potentially any outcome involving increased T cell activation could be due to fewer cancer cells and less soluble inhibitory factor present over the course of the 24 h assay. Enumeration of E.G7 cells did not yield a statistical difference between the cells grown in Static and SMG conditions for 72 h (Fig. 3a). To check the cells for apoptosis, 7-aminoactinomycin D (7-AAD) was added to the Static and SMG cultures during the last 15 min of incubation. The fluorescent dye, 7-AAD, is unable to penetrate the membranes of viable cells but diffuses readily into cells undergoing apoptosis. Again, there was no statistical difference in percentages of apoptosis between E.G7 cancer cells grown in Static (2.2% 7-AAD^+^) and SMG (2.4% 7-AAD^+^) conditions (Fig. 3b). Thus, E.G7 cancer cells grow equally well in both Static and SMG culture conditions over the 72 h incubation period. To assess the effect of SMG upon dendritic cell activation of the T cells, E.G7 cells were first cultured in SMG or Static conditions for 72 h, then the cells were harvested, washed and incubated with JAWS II DC, OT-II TCH and OVA323 for 24 h in Static conditions. JAWS II DC were capable of activating the CD4^+^ T cells to produce significant levels of IL-2 when co-cultured with SMG E.G7 cancer cells as compared to co-culture with Static E.G7 (Fig. 3c). Restoration of IL-2 levels to that of T cells activated in the absence of E.G7 was not achieved. That is, CD4^+^ T cell IL-2 production in the presence of SMG E.G7 remained significantly less (*p* ≤ 0.02) than cytokine production by the T cells activated in the absence of the tumor. Therefore, SMG did reverse the inhibition imposed by E.G7 cells upon DC allowing for T cell activation with a significant 2.5-fold increase in IL-2 secreted.

**Figure 4:**
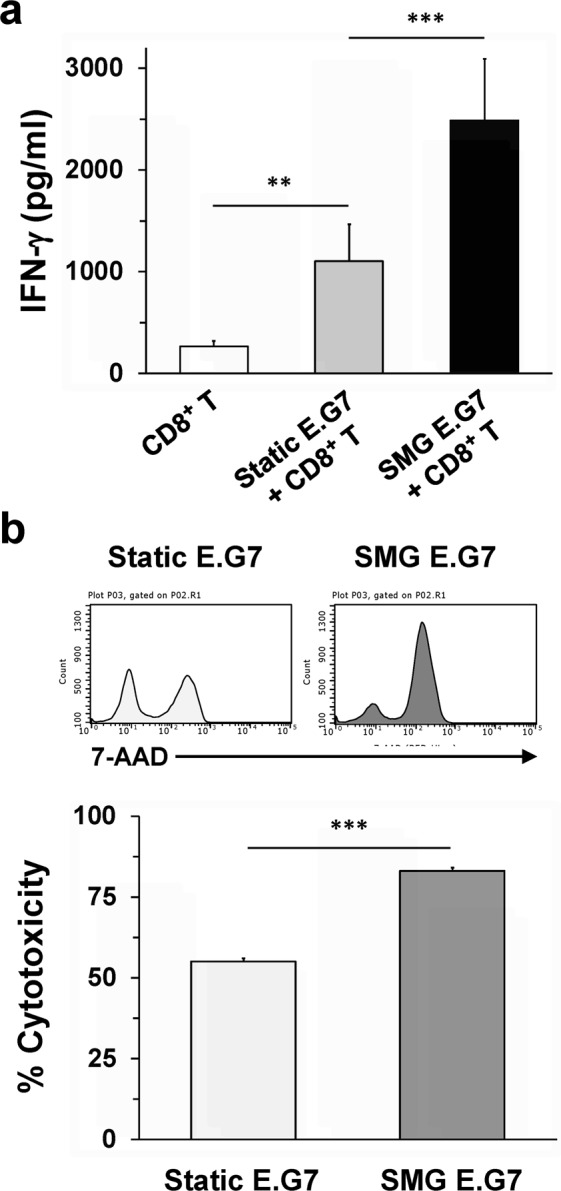
SMG enhances CD8^+^ T cell activation and cytotoxic function. (**a**,**b**) E.G7 tumor cells (2 × 10^5^/ml) were cultured in Static (light gray bar) or SMG (dark gray and black bars) conditions for 72 h. Following culture, Static and SMG E.G7 were collected and incubated (1 × 10^5^/well) separately with OT-I CD8^+^ T cells (1 × 10^5^/well) for 4 or 24 h. Production of IFN-γ by the CD8^+^ T cells (**a**) was assessed by ELISA from supernatants collected after 24 h co-incubation with the tumor cells. In (**b**), Static and SMG E.G7 tumor cells were labeled with CFSE prior to the addition of the T cells. During the last 15 min of a 4 h co-culture, 7-AAD was added to the wells. Then, the cells were analyzed by flow cytometry gating on CFSE^+^ cells to determine the expression of 7-AAD in the labeled Static and SMG E.G7 cells (b, top panel). Percent cytotoxicity was determined by evaluating the number of CFSE^+^ 7-AAD^+^ cells within the total CFSE^+^ population (×100%) (b, bottom panel). For each bar, the data represent the mean + SD of quadruplicate samples of two independent experiments. **p-value ≤ 0.001 comparing CD8^+^ T cell IFN-γ production when cultured with or without Static E.G7 cells. ***p-value ≤ 0.0001 comparing CD8^+^ T cell IFN-γ production or cytotoxicity when cultured with Static and SMG E.G7 cells.

Interestingly, CD8^+^ T cells can be activated by any cell in the body that expresses an MHC class I molecule bound with peptide and recognized by the TCR^7^. In this way, any cell infected by an infectious agent or rendered abnormal due to a transforming event can be effectively detected and eliminated by cytotoxic CD8^+^ T cells. While we investigated the effect of SMG upon dendritic cell activation of T cells above, it was also of interest to determine if SMG could enhance the activation of T cells when the cancer cells were themselves serving as the peptide presenting cell. The E.G7 cell line was generated from the EL-4 parent to express MHC binding peptides from the stably transfected OVA protein in order to assess OVA-specific CD8^+^ T cell responses to a peptide derived from the tumor^10^. Peptide specificity of the CD8^+^ T cells and ability of the E.G7 lymphoma cells to display the proper peptide was confirmed by the 5-fold increase in IFN-γ production by OT-I CD8^+^ T cells when cultured with Static E.G7 cells as compared to CD8^+^ T cells alone (Fig. 4a). The background IFN-γ production by the OT-I CD8^+^ T cells was most likely due to the 7 day culture with IL-2, however, the presence of peptide (OVA protein amino acids 257–264), bound to MHC class I on the surface of E.G7 cells, significantly increased output of IFN-γ (Fig. 4a, white and gray bars). More important, SMG culture of the E.G7 lymphoma cells for 72 h enhanced the responsiveness of the CD8^+^ T cells (Fig. 4a, black bar). Next, we examined whether SMG affected the anti-cancer cytotoxic function of the CD8^+^ T cells. Upon recognition of a cancer cell, CD8^+^ T cells are activated to release cytotoxins locally, which induce apoptosis of the target cell. 7-AAD was added to the co-cultures of Static or SMG E.G7 cells and OT-I CD8^+^ T cells to identify apoptotic E.G7 cancer cells. Viable E.G7 cells were unable to take up 7-AAD while disrupted cell membranes of apoptotic E.G7 permitted intercalation of the dye within the DNA and fluorescence. In order to discriminate 7-AAD^+^ populations as E.G7 and not CD8^+^ T cells, the lymphoma cells were pre-labeled with the membrane-permeable cytoplasmic dye, 5-(6)-carboxyfluorescein diacetate succinimidyl ester (CFSE). Using the flow cytometer, events captured were assessed for the fluorescence of 7-AAD within the total CFSE^+^ population of E.G7 cancer cells (Fig. 4b, top panel). SMG culture of E.G7 cancer cells resulted in a greater increase in the number of apoptotic CFSE^+^ 7-AAD^+^ cancer cells compared to incubation in Static conditions. Indeed, of the E.G7 cancer cells incubated with the CD8^+^ T cells, 83% of the SMG E.G7 cells were apoptotic as compared to only 55% of the Static E.G7 (Fig. 4b, bottom panel). Thus, SMG alters the interaction between E.G7 cancer cells and OVA-specific CD8^+^ T cells leading to a more robust direct activation as indicated by elevated IFN-γ production and percentages of apoptotic E.G7 cells.

### Increased immunogenicity of SMG E.G7 cancer cells translates into improved tumor control

**Figure 5:**
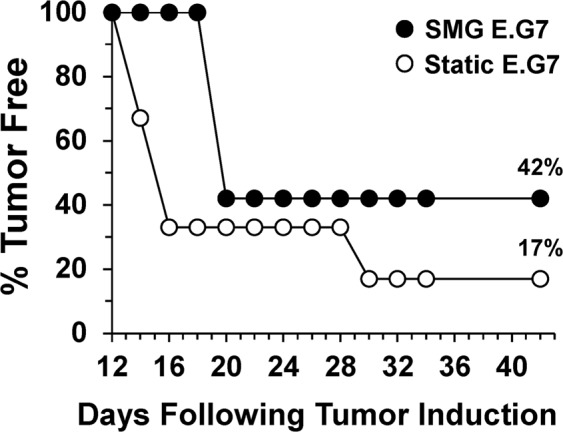
Augmentation of E.G7 tumor control mediated by SMG. E.G7 lymphoma cells (2 × 10^5^/ml) were cultured in Static (white circles) or SMG (black circles) conditions for 72 h. Following culture, Static or SMG E.G7 (1 × 10^6^ in 100 μl PBS/mouse) were collected and injected subcutaneously into C57Bl/6 mice. Tumor presence was palpated every other day and confirmed using a caliper up to 42 days post-tumor induction. Each circle represents the percentage of mice tumor-free at each time point. The data is representative of two independent experiments with each circle expressing the percentages as calculated from an n = 12.

SMG culture of E.G7 lymphoma cells improved T cell cytokine production and killing of the tumors as compared to Static controls. The next question addressed was whether the improvements in reactivity towards the cancer cells were translatable to *in vivo* immune control of tumor development. E.G7 cancer cells were incubated in Static or SMG conditions for 72 h, then the cells were harvested, resuspended in PBS and subcutaneously injected into groups of mice at the nape of the neck. Mice were then monitored by palpation and caliper measurement for tumor development every other day. Tumors were detectable by palpation at 4 mm^2^ and confirmed as a progressively growing neoplasm with two successive increases in size upon caliper measurement. Consistent with other groups, Static cultured E.G7 cancer cells produced progressive tumors in >80% of mice injected^11^. However, only 58% of mice injected with SMG E.G7 cancer cells developed tumors within the clinical observation time (Fig. 5). In addition, SMG incubation of E.G7 cells led to a near week delay in tumor formation in those mice that did eventually develop a tumor. Following tumor development at day 20 in the SMG E.G7 challenged animals, no further mice had detectable tumors throughout the remainder of the clinical assessment. However, the group of mice injected with Static E.G7 cells continued to demonstrate animals that were positive with tumor at time points beyond day 20. Thus, those mice protected from tumor development at day 20 post-tumor induction, likely had eradicated the cancer cells early after introduction into the animals. Overall, SMG exposure for 72 h altered the immunogenicity of E.G7 cancer cells likely involving a more robust T cell response at the outset resulting in the elimination of the abnormal cells so that no lymphoma cells remained to establish a tumor microenvironment.

## Discussion

A number of studies have shown that exposure to a microgravity or SMG environment altered the morphology, signaling, function and growth rate of differing cell types^13,14^. For instance, the human breast cancer cell line MCF-7 was shown to undergo changes in morphology due to alterations of the cytoskeleton and focal adhesions in SMG^15^. Human U-251 glioma cells cultured in SMG were shown to arrest in the G2/M phase of the cell cycle and after 72 h, a third of the population underwent apoptosis^16^. Interestingly, human follicular thyroid cancer cells (FTC-133) flown onboard the Shenzhou-8 spacecraft were found to form multicellular tumor cell spheroids in the cultures^17^. Incubation of the FTC-133 cells in a 2D-clinostat and RPM on Earth also generated spheroids^18^. These scaffold-free spheroids likely resemble an *in vivo* growing tumor as compared to the monolayer cultures currently used in many studies. Formation of spheroids in the 2D-clinostat and RPM resulted in differential modulation of genes such as *ERK1* and *EGF* as well as cytokines including IL-6, IL-8 and macrophage inflammatory protein-1 alpha. While thought to be involved in the process of spheroid formation, connective tissue growth factor (CTGF) was also upregulated in FTC-133 cells when cultured in either the clinostat or RPM. Interestingly, CTGF has been shown to promote both pro-tumor and anti-tumor activities, depending upon the tumor examined^19^. Examination of the E.G7 cancer cells growing in the RCCS also demonstrated some spheroid-like growth patterns, although, during media replenishment, the aggregates were separated by pipetting. Potentially, SMG-mediated spheroid formation may have activated the production of CTGF leading to the inactivation of signaling involved in the production of the inhibitory soluble factor by the E.G7 cancer cells. Spheroid formation and its impact upon tumor evasion will be investigated in future work with the E.G7 lymphoma. Hence, there have been a number of studies examining the impact of microgravity upon tumor growth characteristics but few testing the proposed microgravity-induced reduction in tumorigenesis.

Long-term exposure to space radiation has remained of great concern to the future of human space exploration beyond Earth orbit^1^. More specifically, the potential for radiation-induced stress and damage which can lead to transformation and cancer. Certainly, efforts to shield astronauts from ionizing radiation are ongoing. However, if transformations do occur in astronauts, are the cancer cells more susceptible to the host immune response? If this is the case, then that information may be translated into cancer therapies for patients on Earth. This study set out to provide an answer to the first question and other studies to follow addressing the latter statement. Here, we evaluated the impact of SMG upon the tumor evasion mechanism employed by a murine lymphoma line. Previous work had demonstrated a cell contact-dependent mechanism used by E.G7 cancer cells to reduce CD8^+^ T cell responses^20^, however, we described an evasion tactic employed by E.G7 cells to diminish the function of DC in activating peptide-specific CD4^+^ T cells. Given the cell contact-dependent mechanism eluded to above was shown to dampen CD8^+^ T cell responses directly, likely our findings related to increased CD8^+^ T cell responses involved disruption of this evasion tactic. While the specific mechanism(s) of E.G7 cell evasion of both CD4^+^ and CD8^+^ T cells was not the scope of the investigation, future work will elucidate these pathways and confirm their modulation by SMG. Notwithstanding, E.G7 lymphoma cells were shown for the first time to reduce dendritic cell activation of peptide-specific CD4^+^ T cells through a soluble factor rather than a cell contact-dependent mechanism. We assessed the co-culture supernatant for the best known tumor-derived inhibitory soluble factors, transforming growth factor-beta (TGF-β), interleukin-10 (IL-10) and vascular endothelial growth factor (VEGF)^21^. E.G7 tumor cells cultured for 72 h in Static and SMG conditions produced less than 100 pg/ml of IL-10 and VEGF each with no substantial TGF-β detected in the culture wells (data not shown). In addition, no statistical difference was found when comparing IL-10 and VEGF levels between the Static and SMG E.G7 cells. Therefore, the soluble factor responsible for suppressing DC in this model system remained undefined and will be the subject of future investigation.

Interestingly, the culture of E.G7 cancer cells in the RCCS for 72 h resulted in an apparent disruption of tumor evasion function as JAWS II DC were able to promote IL-2 production by the CD4^+^ T cells. However, the level of IL-2 was not restored to that of T cells activated by DC in the absence of E.G7 cells. Thus the action of SMG to reduce tumor evasion was not a complete reversion, also suggesting that production of the soluble inhibitory factor was only reduced but not eliminated. Despite this, the T cells were able to produce significantly more IL-2 than when the DC were co-cultured with SMG E.G7 cells when compared to incubation with Static E.G7 cells. Given CD8^+^ T cells can also be activated by DC, it was likely that stimulation of these T cells would be similarly affected by the inhibition imposed on the DC by the tumor-derived factor. More important, CD8^+^ T cells can interact directly with the tumor cells for activation and effector function. The E.G7 cells were generated from the parent lymphoma EL-4 cell line by transfection with a gene encoding the full-length OVA protein^10^. This permitted the examination of the interactions of OVA peptide-specific CD8^+^ T cells with MHC class I-positive tumor cells in terms of activation and outcomes. E.G7 cells expressed OVA peptide (amino acids 257–264) and activated the OT-I CD8^+^ T cells directly. Given the dependence of CD8^+^ T cell activation upon IL-2 provided by CD4^+^ T cells^22^, it was clear why the OT-I CD8^+^ T cells were activated by the E.G7 cells directly. First, IL-2 had been provided to the CD8^+^ T cells in culture up to the point of E.G7 cell addition, thus, as long as E.G7 cells provided peptide bound to MHC class I molecules, the T cells were able to respond. As mentioned above, E.G7 lymphoma cells produce a cell surface molecule employed to suppress CD8^+^ T cell responses. E.G7 cancer cells express programmed cell death receptor ligand-1 (PD-L1) on the cell surface^20^. The receptor, programmed cell death-1 (PD-1), can be found on CD8^+^ T cells upon chronic activation^23^. Ligation of PD-1 by PD-L1 has been shown to diminish T cell responses against virally infected cells and cancer cells. The naïve CD8^+^ T cells used in this study were harvested from cryopreservation and expanded with IL-2 for less than a week and during that time were never incubated with peptide and DC. Hence, the T cells likely did not express significant levels of PD-1 to notably impair responses. Albeit, we measured a significant increase of IFN-γ production by the CD8^+^ T cells when co-cultured with SMG E.G7 cancer cells as compared to Static E.G7. Such a response by the T cells would support the notion that the cells have low PD-1 expression given this receptor would likely have blocked or diminished cytokine production^23^. At the same time, chronic IFN-γ has been shown to promote tumor growth primarily through induction of PD-1 expression on T cells and PD-L1 on DC^24^. But as discussed, our CD8^+^ T cells were only incubated for less than a week in the presence of IL-2 and produced low levels of IFN-γ at the time of activation by the tumor, thus, it was highly unlikely that there was sufficient cytokine to promote PD-1 expression in such a short time frame. Alternatively, MHC class I protein expression may have been increased on the surface of the E.G7 cells by SMG exposure. Indeed, we recently observed increased MHC class I expression on JAWS II DC upon SMG culture for 72 h (data not shown). In addition, IFN-γ has been shown to increase MHC class I expression on the surface of tumor cells resulting in an augmentation of CD8^+^ T cell cytotoxicity^21^. In this case, activation of the T cells by SMG E.G7 cells could have produced a feedback loop where increasing IFN-γ resulted in elevated MHC class I expression on the tumor cells and enhanced cytokine production and cytotoxicity. Therefore, SMG produced a dramatic influence upon CD8^+^ T cell cytokine production and cytotoxicity against tumor cells. This most likely involved an alteration of the primary evasion mechanism employed by tumors to escape T cell cytotoxicity, namely, reduced MHC class I expression.

We further tested whether SMG could enhance immune responses against a tumor *in vivo*. Previously, B16-F10 murine melanoma cells were placed in an RCCS for 48 h and then inoculated subcutaneously into mice. Proliferation of SMG cultured B16-F10 cells was reduced compared to Static B16, however, tumor growth was significantly increased^25^. This would suggest that despite a negative impact upon B16 melanoma proliferation *in vitro*, SMG may have enhanced tumorigenicity of B16 and perhaps E.G7 cells *in vivo*. Interestingly, we did not demonstrate a negative impact of SMG upon E.G7 cancer cell growth or viability, indicating that any modulation of tumor development most likely would be due to altered tumor evasion. Striking, mice inoculated with SMG E.G7 cells demonstrated superior tumor control to that of animals injected with Static E.G7 cells. Such a discrepancy must have been due to the soluble factor expressed by E.G7 lymphoma cells as tumor evasion mediated by B16 melanoma was known to involve the production of IL-10^26^, TGF-β and VEGF^27^ as well as surface expression of programmed cell death ligand 1 (PD-L1)^28^. The E.G7 cancer cells did not produce significant levels of any of the noted suppressive cytokines above (data not shown) however, as noted above, the E.G7 lymphoma cells have been previously shown to express PD-L1^20^. From our study, E.G7 tumor evasion of CD4^+^ T cell activation was primarily due to a secreted factor and not a cell contact-dependent mechanism that acted on the JAWS II DC. For B16-F10 melanoma cells, although not identified, likely the reduced tumor control *in vivo* was related to increased inhibition of T cell responses from either elevated cytokine production (IL-10, TGF-β) or ligation of PD-1. For E.G7 cells, SMG diminished the inhibitory action of the soluble factor *in vitro* which likely translated to improved T cell cytokine responses *in vivo*. In addition, SMG improved CD8^+^ T cell cytokine production when interaction occurred between tumor and T cell. Furthermore, SMG E.G7 cells were more susceptible to CD8^+^ T cell cytotoxicity than Static E.G7 cells. Both findings suggest that SMG increased the interaction of the CD8^+^ T cell TCR with peptide/MHC I in terms of activation signaling for cytokine production and recognition for cytotoxin release. Thus, SMG most likely increased MHC class I molecules on the surface of E.G7 cells to enhance CD8^+^ T cell responsiveness. Overall, SMG diminished E.G7 cell escape from T cell responses leading to improved tumor control *in vivo* as compared to E.G7 cells cultured in Static conditions. Detailed examination of the different evasion mechanisms in tandem with SMG may bring to light evasion pathways critical to the survival and/or aggressiveness of a wide variety of tumor cell types including previously unknown mechanisms as demonstrated here for E.G7 lymphoma cells. In turn, this information could be used to devise new therapeutic approaches to best disrupt tumor growth and improve tumor control and eradication by the immune system.

## Methods

### Cells

Murine JAWS II DC and E.G7-OVA tumor cells were obtained from the American Type Culture Collection (ATCC, Manassas, VA). JAWS II DC were maintained in alpha minimum essential medium (+ribonucleosides and deoxyribonucleosides) supplemented with 20% fetal bovine serum, 5 ng/ml recombinant murine GM-CSF (BD Biosciences) and gentamicin (Sigma-Aldrich). E.G7-OVA (E.G7) was a derivative of the parent T cell lymphoma line EL-4, stably transfected to constitutively express chicken ovalbumin (OVA). As such, E.G7 tumor cells were maintained in RPMI-1640 media (ATCC) with 10% FBS and supplemented with gentamicin, 50 μM β2-mercaptoethanol or BME (Sigma-Aldrich) and 400 μg/ml neomycin (ThermoFisher). The CD4^+^ T cell hybridoma (TCH) line, BO97.10.5 (OT-II TCH) was a kind gift from Philippa Marrack, National Jewish Health, Denver, CO, USA. OT-II TCH were sustained in culture with 10% FBS in Spinner modification-minimum essential media (Sigma-Aldrich) and added T cell line cocktail (ThermoFisher) composed of: dextrose, glutamine, 50X essential amino acids, 100X non-essential amino acids, sodium bicarbonate, gentamicin, penicillin G, streptomycin sulfate, 50 μM BME and sodium pyruvate adjusted to pH 7.0. The OT-II TCH respond specifically to ovalbumin (OVA) peptide, amino acids 323–339, or OVA323 (InvivoGen) when displayed by DC (I-A^b^-restricted). OT-I CD8^+^ T cells (H2-K^b^-restricted) were generated from harvested spleens and lymph nodes of 8- to 12-week-old OT-I T cell receptor transgenic C57Bl/6 mice (Charles River Laboratories). The harvested cells contained both T cells and DC so that peptide can be added to expand the CD8^+^ T cells. Subsequently, OVA peptide, amino acids 257–264 (InvivoGen), was added (10 μg/ml) along with IL-2 (50 ng/ml) (R&D Systems) to the cells bathed in medium containing RPMI-1640 with 10% FBS, 50 μM β2-mercaptoethanol (Sigma Aldrich) and gentamicin for 3 days. Afterward, the enriched CD8^+^ T cell population was cryopreserved in liquid nitrogen^29^. For experimentation, the T cells were thawed in medium + IL-2 (25 ng/ml), then refreshed with media and cytokine on day 5. The CD8^+^ T cells were used on day 7 with a consistent purity of 95% when assessed by flow cytometry (data not shown). All cells were maintained at 37 °C in a humidified atmosphere of 5% CO_2_ and 95% air.

### Mice

C57Bl/6 mice were purchased from Charles River Laboratories and housed in the animal facility at Converse College. OT-I T cell receptor transgenic C57Bl/6 mice were purchased from Charles River Laboratories and sacrificed upon delivery to generate CD8^+^ T cells as outlined above. All procedures and experiments involving these animals were approved by the Converse College Institutional Animal Care and Use Committee (IACUC). In addition, all methods and experiments were performed in accordance with the relevant guidelines and regulations established by the Converse College IACUC.

### Rotary cell culture system (RCCS)

The rotary cell culture system (Synthecon Inc.) was developed by NASA to investigate the effects of SMG upon cells on Earth^30^. E.G7 lymphoma cells were added to a rotation vessel (RV) in media at a concentration of 2 × 10^5^/ml (10 ml total volume). Subsequently, all air was removed from the RV before vertical attachment to the RCCS. Gas exchange with the environment was accomplished through the silicon membrane lining the bottom of the RV. The loaded rotator device of the RCCS was placed in the CO_2_ incubator and cell rotation adjusted to 16 rpm. This rotation speed was selected based upon the successful culture of other murine cancer cell lines in our lab, set-up guidelines of the manufacturer, optimal rotation of the E.G7 cells with clear cell aggregation in the RV center and minimal cell death, as measured by Trypan exclusion and flow cytometry, and similar growth results for other cells rotated at this speed in the literature. At this speed, the cells remained in the center of the RV where fluid shear force is minimal creating a state of sustained free-fall or SMG. RV containing E.G7 tumor cells were removed briefly from the RCCS after 48 h in order to replenish media for the additional 24 h of rotation (72 h total rotation). The media is changed every 48 h in order to prevent growth inhibition due to the buildup of waste products and the reduction of nutrients. Control E.G7 tumor cells were cultured in 25 cm^2^ flasks (10 ml total volume) in the incubator next to the RCCS rotation device. Preliminary work comparing the growth of E.G7 in 25 cm^2^ flask versus a stationary RV yielded no significant difference in assay outcomes thereby validating the use of flasks to reduce the cost of experimentation. Cells cultured in the flasks are growing in Static conditions.

E.G7 tumor cells cultured in Static or SMG conditions were designated in all experimentation as Static E.G7 and SMG E.G7 cells, respectively.

To determine if SMG affects the growth and viability of E.G7 lymphoma cells, E.G7 cells (2 × 10^5^/ml) were cultured in SMG or Static conditions for 72 h. Following harvest from the RV and control flask, the cells were enumerated by Trypan exclusion and assessed for apoptosis by 7-aminoactinomycin D (7-AAD, BD Biosciences) staining and flow cytometric analysis. 7-AAD was excluded from viable cells but able to bind DNA in apoptotic cells and upon interaction with the nucleic acid undergo a spectral shift. 7-AAD was detected in the FL3 channel (red) of the flow cytometer. Apoptotic E.G7 tumor cells were 7-AAD^+^ while viable E.G7 cells were 7-AAD^−^. Percentages of 7-AAD^+^ E.G7 cells were calculated by dividing the number of 7-AAD^+^ cells with the total population, including the 7-AAD^−^ E.G7 cells (×100%).

### E.G7 lymphoma cell and JAWS II DC co-culture

For the assay involving removal of E.G7 cells prior to T cell addition, initially, JAWS II DC (5 × 10^4^) were incubated with Static E.G7 cancer cells (2.5 × 10^4^) for 24 h in a 24-well plate. Cultures of JAWS II DC previously demonstrated that 80% of the cells adhered to the flask surface while 20% remained in suspension. E.G7 cells remained in cell suspension without any adherence whatsoever. Following the 24 h co-culture, E.G7 tumor cells were removed from the wells and the adherent JAWS II DC were gently washed and then replenished with fresh media. T cells and peptide were then added as outlined below.

For the experiment involving separation of the cancer cells and DC, E.G7 tumor cells (2.5 × 10^4^) were added to the upper chamber of a 24-well plate containing a Transwell insert (Millipore) with a pore size of 0.4 μm. JAWS II DC (5 × 10^4^) were added to the lower chamber of the same well and co-incubation continued for 24 h. Then, the insert and E.G7 cells were removed from the wells and T cells and peptide added as noted in the figure.

In order to establish co-cultures of SMG E.G7 cancer cells and DC, E.G7 cells (2 × 10^5^/ml) were added to an RV and rotated at 16 rpm for 72 h. Control Static E.G7 cells (2 × 10^5^/ml) were cultured in a 25 cm^2^ flask within the same incubator next to the rotation device. Static and SMG E.G7 tumor cells were harvested and added (2.5 × 10^4^/well) to separate wells of a 24-well plate containing JAWS II DC (5 × 10^4^) and incubated for 8 h. This time point was chosen to allow for interaction between the SMG E.G7 cells and JAWS II DC without recovery of E.G7 suppressive function prior to the addition of T cells and peptide.

### T cell activation assays

For CD4^+^ T cell activation, OT-II TCH (1.25 × 10^5^/well) were added to wells containing JAWS II DC (5 × 10^4^/well) and either Static or SMG E.G7 tumor cells (2.5 × 10^4^/well). Furthermore, OVA323 (0.1 mg/ml) was included in some culture wells. In certain experiments, the E.G7 cells were removed prior to the addition of the T cells and/or peptide. Once all cells and reagents were added, incubation continued for a total of 24 h.

For CD8^+^ T cell activation, OT-I CD8^+^ T cells (1 × 10^5^/well) were added to wells (96-well plate) containing the Static or SMG E.G7 tumor cells (1 × 10^5^/well) and incubated for 24 h in order to measure production of IFN-γ. No DC were included in this culture as it was previously shown that E.G7 tumor cells produce and display the peptide for which OT-I CD8^+^ T cells respond^29^.

### Detection of T cell production of IL-2 and IFN-γ

IL-2 and IFN-γ production by T cells was detected using the BD Biosciences recommended ELISA protocol. CD4^+^ T cell production of IL-2 was captured from supernatants using rat anti-mouse IL-2 (JES6–1A12) and detected using biotinylated rat anti-mouse IL-2 (JES6–5H4) antibodies. Whereas CD8^+^ T cell production of IFN-γ was measured in supernatants using rat anti-mouse IFN-γ (R4–6A2) and biotinylated rat anti-mouse IFN-γ (XMG1.2) antibodies. Graded amounts of recombinant murine IL-2 and IFN-γ were included in the assays for generation of standard curves from which reported concentrations were extrapolated using the linear portion of the curve. The IL-2 and IFN-γ concentrations were acquired using a BioTek Eon microplate spectrophotometer (BioTek Instruments Inc.) and analyzed using Gen 5 software version 2.01.14.

### CD8^+^ T cell-mediated cytotoxicity of E.G7 tumor cells

Static and SMG E.G7 tumor cells were harvested following 72 h incubation in a 25 cm^2^ flask and RV, respectively. The cells were individually counted and resuspended at a concentration of 1 × 10^6^ viable cells in PBS. The cells are then labeled with 5 μM of CellTrace™ 5-(6)-carboxyfluorescein diacetate succinimidyl ester (CFSE, ThermoFisher) for 20 min. This green probe diffused into the cells and was cleaved by intracellular esterases to produce a highly fluorescent molecule detected in the FL1 channel (green) of the flow cytometer. The cells were washed and adjusted to a concentration of 1 × 10^5^ cells and added in quadruplicate to wells of a 96-well plate. Unlabeled OT-I CD8^+^ T cells were generated as discussed above and added to the wells containing CFSE-labeled Static and SMG E.G7 tumor cells at a concentration of 1 × 10^5^ cells/well. The co-culture of tumor and T cells was then placed in the incubator for 4 h. 15 min prior to the end of the 4 h assay, 12.5 μg of 7-AAD was added to each well in the assay plate. Upon completion of the 4 h incubation, the co-cultures were analyzed by a Millipore Guava Cytometer (EMD Millipore) with 50 × 10^3^ events collected and analyzed with GuavaSoft 2.7 software. Apoptotic E.G7 tumor cells were CFSE^+^ 7-AAD^+^ while viable E.G7 cells were CFSE^+^ 7-AAD^−^. Histograms were generated by gating on the CFSE^+^ population to reveal the 7-AAD^+^ and 7-AAD^−^ E.G7 cells.

Percentages of CD8^+^ T cell cytotoxicity were calculated by dividing the number of CFSE^+^ 7-AAD^+^ E.G7 cells with the total number of CFSE^+^ events (×100%). Given CFSE is removed from the E.G7 cells prior to CD8^+^ T cell addition, cells detected as CFSE^+^ are E.G7 cells. Percentages of Static and SMG CFSE^+^ 7-AAD^+^ E.G7 cells cultured without OT-I CD8^+^ T cells during the 4 h assay were subtracted from the final percentages calculated to account for background apoptosis in the population.

### E.G7 tumor growth *in vivo*

Following culture of E.G7 tumor cells in Static and SMG conditions, cells were collected, washed with PBS, counted and viable cells resuspended at a concentration of 10 × 10^6^ cells/ml. C57Bl/6 mice (8–10 weeks of age) were subcutaneously injected with 100 μl of Static or SMG E.G7 tumor cells (1 × 10^6^ cells/mouse) at the nape of the neck. Palpation for tumor growth was assessed every other day and confirmed with subsequent detection with a Vernier caliper for two consecutive measures of increased growth. Mice without a detectable and confirmed tumor were assigned the designation of tumor-free. Mice were sacrificed using approved IACUC methods upon tumor growth of 200 mm^2^ or at the end of the clinical observation of 42 days post-tumor induction.

### Statistical analyses

Results were expressed as shown in each figure with bar graphs generally represented as means of duplicate to triplicate experiments and each test sample performed at a range of n = 4–12. Differences between group means were calculated for statistical significance using the unpaired *t*-test with p < 0.05 considered as a significant difference. All *t*-test calculations were performed using GraphPad software (GraphPad Software Inc.).

## Data Availability

The harvest, culture and cryopreservation of OT-I CD8^+^ T cells were previously published as referenced. The culture of cells in the RCCS or flasks is discussed above and any further information can be made available by the corresponding author. E.G7-OVA and JAWS II DC were obtained from ATCC. All relevant data are included in this article.
